# Gene *emrC* Associated with Resistance to Quaternary Ammonium Compounds Is Common among *Listeria monocytogenes* from Meat Products and Meat Processing Plants in Poland

**DOI:** 10.3390/antibiotics13080749

**Published:** 2024-08-09

**Authors:** Iwona Kawacka, Agnieszka Olejnik-Schmidt

**Affiliations:** Department of Food Biotechnology and Microbiology, Poznan University of Life Sciences, Wojska Polskiego 48, 60-627 Poznan, Poland

**Keywords:** food safety, disinfection, genotypes, gene profiles, virulence, cross-resistance

## Abstract

(1) Background: *L. monocytogenes* is a food pathogen of great importance, characterized by a high mortality rate. Quaternary ammonium compounds (QACs), such as benzalkonium chloride (BC), are often used as disinfectants in food processing facilities. The effectiveness of disinfection procedures is crucial to food safety. (2) Methods: A collection of 153 isolates of *L. monocytogenes* from meat processing industry was analyzed for their sensitivity to BC using the agar diffusion method. Genes of interest were detected with PCR. (3) Results: Genes *emrC*, *bcrABC,* and *qacH* were found in 64 (41.8%), 6 (3.9%), and 1 isolate (0.7%), respectively, and 79 isolates (51.6%) were classified as having reduced sensitivity to BC. A strong correlation between carrying QACs resistance-related genes and phenotype was found (*p*-value < 0.0001). Among 51 isolates originating from bacon (collected over 13 months), 48 had the *emrC* gene, which could explain their persistent presence in a processing facility. Isolates with the *ilsA* gene (from LIPI-3) were significantly (*p*-value 0.006) less likely to carry QACs resistance-related genes. (4) Conclusions: Reduced sensitivity to QACs is common among *L. monocytogenes* from the meat processing industry. Persistent presence of these bacteria in a processing facility is presumably caused by *emrC*-induced QACs resistance.

## 1. Introduction

### 1.1. Listeria Monocytogenes and Food Safety

Food is essential to life, and owing to this fact, food safety is considered to be a basic human right [1]. The major challenges of providing non-harmful food include its microbiological safety, especially in the context of bacteria, as they cause more foodborne incidents than other microbe groups, e.g., viruses or protozoa. Bacterial genera responsible for most foodborne infections include *Salmonella*, *Vibrio*, *E. coli*, *Shigella*, *Brucella*, *Campylobacter*, and *Listeria* [1]. Among those foodborne pathogens, *Listeria monocytogenes* presents the highest death toll, estimated to reach approximately 13–15% in the United States [2,3], or even up to 20–30% for those who contract listeriosis [4,5,6,7]. These bacteria are responsible for 19% of the total deaths caused by consumption of contaminated food products in the USA [8], and due to high mortality and high hospitalization rate of affected patients, *L. monocytogenes* causes tremendous annual economic losses, reaching almost USD 2.8 billion. That constitutes 18% of the total economic burden caused by major foodborne infectious agents associated with illnesses acquired through food products [2,3].

*L. monocytogenes* is a ubiquitous bacterium which can be found in water, soil, decaying vegetation, and even the human digestive tract [9]. It is characterized by easy adaptation to environmental conditions, including its ability to grow in a wide range of temperatures (0 °C–45 °C) and pH (4.3–9.6), toleration of high salt concentrations (up to 10.0% NaCl), and low water activity (A_w_ to 0.90) [6]. These features facilitate survival and multiplication in food processing facilities [5], leading to its wide distribution and, in some cases, persistence in food processing environments [10]. Foods associated with high rates of *L. monocytogenes* infections include raw and processed food of both animal and plant origins, for example: raw sprouts, unpasteurized milk, soft cheeses, cold deli meats, cold hot dogs, as well as smoked seafood [9].

The presence of *L. monocytogenes* in food products is strictly regulated in many countries. In the United States, absence of *L. monocytogenes* in ready-to-eat food is required, whereas in European Union, as well as in Canada and Australia/New Zealand, absence is required in foods intended for infants and special medical purposes. In other foods, the bacteria may not exceed 100 CFU/g throughout its shelf-life [11]. Hence, effective and efficient elimination of *L. monocytogenes* from food processing environments is crucial in order to comply with legal regulations and to provide safe food products. Quaternary ammonium compounds (QACs), such as benzalkonium chloride (BC), are widely used for disinfection procedures in food processing facilities due to their efficacy against a variety of bacteria, fungi, and viruses; noncorrosive properties; and biodegradability [12,13,14,15].

However, resistance of *L. monocytogenes* to QACs can occur, as sanitizers may exert selective pressure, causing QACs-resistance associated genes to be maintained or acquired [5]. Among those genes, there are *bcrABC, qacH*, *emrE*, *emrC*, *qacC*, and *qacA*, which have been identified as the most common in the genomes of *L. monocytogenes* in the USA [16]. Proteins encoded by these five genes all belong to the small multidrug resistance family [12,14,17,18,19]. However, other factors, such as *mdrL* [20], which, on the other hand, falls into the major facilitator superfamily [17], or *fepR* [21], which is a transcriptional regulator [22], have also been identified as being involved in diminished BC sensitivity.

Furthermore, not only does QACs resistance reduce the effectiveness of disinfection procedures, but it is also a proven factor leading to cross-resistance of *L. monocytogenes* to antibiotics, such as chloramphenicol, ciprofloxacin, clindamycin, kanamycin, novobiocin, penicillin, streptomycin, and trimethoprim [23,24,25]. Hence, the cross-resistance phenomenon poses a threat to the effectiveness of antibiotic treatment of listeriosis. On the other hand, *L. monocytogenes* isolates well-adapted to food-processing environments, characterized by higher occurrence of genes involved in stress resistance and tolerance to disinfectants, are usually hypovirulent and less likely to cause an infection [26].

### 1.2. Aim of the Study

The main aim of this study was to analyze the occurrence of common genes responsible for resistance to QACs (*bcrABC*, *emrC,* and *qacH*) among 153 *L. monocytogenes* isolates originating from meat products and meat processing plants in Poland and to find differences between the isolates in the collection in terms of their sensitivity to BC.

The additional aim of this study was to correlate presence of Listeria Pathogenicity Island 3 (LIPI-3) in the genomes of collected bacteria to the QACs sensitivity phenotype and QACs resistance-related genes in order to verify a hypothesis of diminished virulence potential among QACs-adapted isolates.

## 2. Results

**Figure 1:**
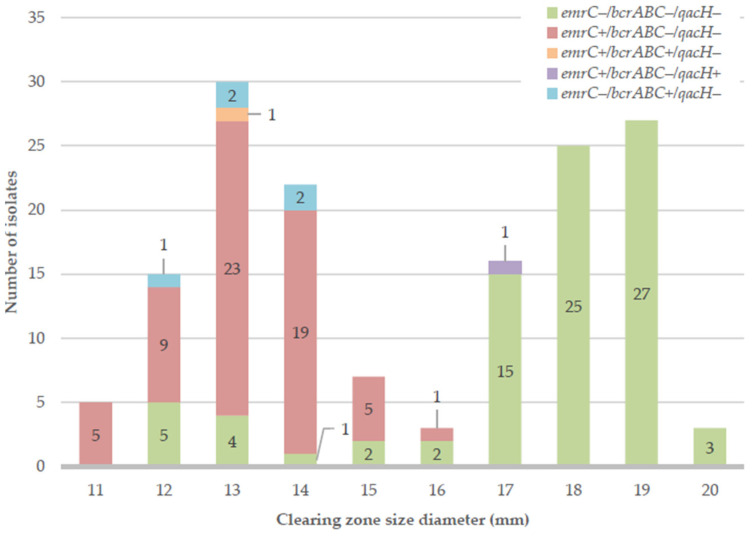
Histogram showing the distribution of the diameters of clearing zones around the BC solution (6 mg/mL) drop along with gene profile information.

The results of sensitivity testing of the isolates to BC are presented in Figure 1 in the form of a histogram. The histogram shows the distribution of particular sizes of the clearing zones achieved around the spot of BC solution (6 mg/mL) placement in an agar diffusion assay. Additionally, five gene profiles achieved for the three QACs-resistance associated genes are indicated on the bars with colors, according to the legend.

In the histogram, there is a clear bimodal distribution, indicating the existence of two subpopulations within the collection of isolates. The subpopulation represented by the bars on the left side of the chart (from 11 to 15 or 16 mm) is characterized by a reduced susceptibility to BC compared to the subpopulation on the right side (from 16 or 17 mm to 20 mm). Based on the histogram distribution, the clearing zone diameter equal to or lower than 15 mm was a criterion applied to characterize isolates with reduced sensitivity to BC. Isolates with a zone diameter equal to or higher than 17 mm were considered sensitive, whereas isolates characterized by a clearing zone diameter of 16 mm were considered to present an intermediate response.

With those criteria applied, more than half, namely, 79 isolates (51.6%) of the total isolates showed reduced sensitivity to BC, which indicates prevalence of reduced sensitivity to QACs among *L. monocytogenes* isolates originating from the food industry. Summarized results of the phenotypic analysis are presented in Table 1.

In terms of genes related to QACs resistance, the gene *emrC* was the most prevalent and was present in 64 isolates (41.8%), followed by the *bcrABC* gene, which was present in 6 isolates (3.9%). The gene *qacH* was present in one isolate (0.7%). Overall, the collection was characterized by five different gene profiles based on the detection of those three genes. The most prevalent gene profile was *emrC−/bcrABC−/qacH−* (represented by green bars in Figure 1), and 84 isolates (54.9%) had that genotype. The second most prevalent was *emrC+/bcrABC−/qacH−* (represented by red bars)*,* and 62 isolates (40.5%) were characterized by that genotype. The gene *bcrABC* alone was detected in five isolates (3.3%) (represented by turquoise bars), and in one isolate (0.7%) together with the *emrC* gene (represented by an orange bar). The gene *qacH* was present in only 1 isolate (0.7%), which also had the *emrC* gene (represented by a purple bar).

The correlation between carrying at least one of the QACs-resistance related genes and the presented phenotype is very strong (chi-square *p*-value < 0.0001). Among 71 isolates considered to be sensitive, 70 isolates (98.6%) did not have any analyzed QACs resistance-related genes. Interestingly, the one remaining isolate (1.4%) carried not only one, but two genes (gene profile *emrC+/bcrABC−/qacH+*), which, on the contrary, would suggest a strong tolerance to QACs. This one isolate (represented by a purple bar in Figure 1) originated from a meat processing environment (conveyor belt).

Among 79 isolates characterized by a reduced sensitivity to BC, 67 (84.8%) had at least one QACs-resistance associated gene, and the remaining 12 isolates (15.2%) did not have any analyzed QACs resistance-related genes. In our earlier studies, we have demonstrated that 10 isolates in this collection were characterized by a reduced sensitivity to ciprofloxacin [27]. Interestingly, all 10 of those isolates fall within the group of those 12 isolates with reduced sensitivity to BC, but which do not harbor any analyzed QACs-resistance related genes. That suggests the existence of a common mechanism for QACs and ciprofloxacin resistance, which is not related to any of genes included in the study herein.

**Figure 2:**
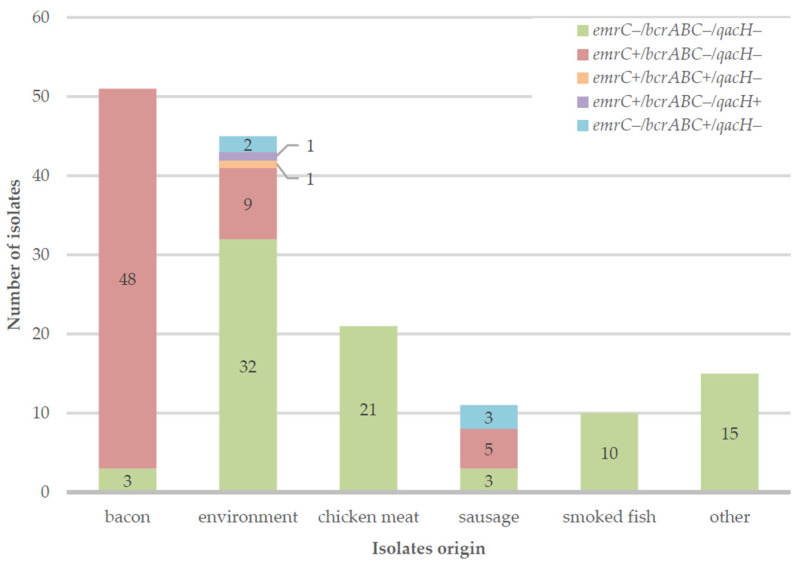
Bar chart showing the sources of the isolates included in the study along with gene profile information.

A clear pattern between an isolate origin and their QACs resistance gene profile can be observed. In the collection, there were 51 isolates originating from bacon (collected over the period of 13 months), and 48 of them had the *emrC* gene. All those 48 isolates had reduced sensitivity to BC in the phenotypic analysis. Resistance to QACs could be a potential reason for the persistent presence of *L. monocytogenes* in bacon samples, as their eradication from the processing plant could be impaired. Apart from bacon samples, 13 environmental samples (out of which 9 originate from sewage drain swabs) and 8 sausage samples had isolates with at least one QACs resistance-related gene. A bar chart showing the sources of the isolates along with their gene profiles is presented in Figure 2.

The *ilsA* gene, the first one from LIPI-3, was the only virulence-associated one included herein, as the collection has been pre-characterized in our previous work. We demonstrated that all 153 isolates harbored at least one variant of 12 analyzed virulence-associated genes, including all from LIPI-1, internalins, and others [28]. The *ilsA* gene analyzed herein was present in 18 isolates (11.8%).

Overall, the analyses of genes included in the study allowed us to differentiate seven groups of isolates with different gene profiles. Summarized results of the genetic analyses are presented in Table 2.

The *ilsA* gene was detected both in isolates without any QACs resistance genes (15 isolates) and in isolates with the *emrC* gene (3 isolates). Isolates carrying the *ilsA* gene were statistically significantly (chi-square *p*-value 0.006) less likely to carry any QACs resistance-related genes. This result supports a hypothesis about diminished virulence potential among QACs-adapted isolates. However, there was no statistical significance between carrying the *ilsA* gene and the presented phenotype (chi-square *p*-value 0.294), as the *ilsA* gene was detected in six isolates classified as sensitive, one isolate classified as intermediate, and eleven isolates with reduced sensitivity to BC.

## 3. Discussion

The most common QACs resistance-associated gene was *emrC*, detected in 64 (41.8%) isolates in this paper. In other studies, this gene was also identified, albeit less frequently. For example, analysis of a set of 25,083 publicly available *L. monocytogenes* genomes from the United States revealed that approximately 0.04% of isolates have that gene [16]. This analysis, however, included samples from various sources, including, e.g., human or aquatic animals, where selective pressure for QACs resistance genes is weaker than in food processing environments, where disinfection procedures are often performed. On the other hand, in an analysis of 4969 genomes of *L. monocytogenes* collected from United States food processing facilities, the gene *emrC* was not detected in any case [29]. However, in the study which included 13 *Listeria* isolates from a meat processing facility in Ireland, the gene *emrC* was present in two persistent isolates and one “presumed non-persistent” *L. monocytogenes* isolate [13]. Similarly, in a study from Poland, where 48 *L. monocytogenes* isolates from ready-to-eat meat and meat processing environments were examined, three isolates harbored *ermC* [30]. These results indicate that *emrC* is more common in Europe than in the United States. Overrepresentation of *emrC* in our collection is probably a result of issues with persistent presence of *L. monocytogenes* in a bacon-producing factory, presumably due to *emrC*-induced QACs resistance.

The gene *bcrABC*, which was present in six isolates (3.9%), was the second most common in our study. In many studies examining QACs resistance determinants, this gene is the most frequently identified. For example, in an already mentioned study which analyzed publicly available *L. monocytogenes* genomes from the United States, the *bcrABC* was identified in as much as 28.6% of samples [16], and in a study which included only food processing samples, it was present in nearly half (46%) of all isolates [29]. In Canadian studies, 41.5% (out of 1279 analyzed) *L. monocytogenes* isolates from various foods and food manufacturing environments harbored the *bcrABC* gene [31]. In Europe, *bcrABC* was identified in 3 out of 13 *Listeria* isolates from an Irish meat processing facility [13] and 1 out of 48 from Polish meat products and meat processing plants [30]. Contrarily to *emrC*, the results indicate more frequent presence of *bcrABC* in the United States than in Europe.

The gene *qacH* was the least frequent and was present in one isolate (0.7%) in our study. In some publications, this gene is reported to be identified very frequently, e.g., in 40% of the *Listeria* isolates from dairy products and the cattle environment in Egypt [32] or even up to 83% of *L. monocytogenes* in strains isolated from fish, fish products, and food-producing factories in Poland [33]. However, studies on bigger sample collections have found that this gene is present in 1.76% of *L. monocytogenes* from United States [16], in approximately 5% of isolates from food processing facilities in the United States [29], and in 1.09% of *L. monocytogenes* contamination in food manufacturing environments in Canada [31]. Hence, the prevalence of *qacH* reported herein is low.

Among the 12 isolates (15.2%) with reduced sensitivity to BC, whose phenotypes were not explained by the detection of any QACs resistance-related genes, there were 10 isolates presenting reduced sensitivity to ciprofloxacin, which have been identified in earlier studies [27]. That suggests a cross-resistance between QACs and ciprofloxacin. Indeed, QACs adaptation is known to increase minimal inhibitory concentrations (MICs) of *L. monocytogenes* to ciprofloxacin, which has already been reported in literature [20,23,24]. Furthermore, to some extent, other genes, such as *emrE*, *qacC*, *qacA*, *mdrL,* and *fepR* [14,16,20,21], may have played a role in the diminished sensitivity of isolates to BC; however, those genes were not analyzed herein.

In terms of phenotypic analysis, in order to determine the response of *L. monocytogenes* to BC, authors usually evaluate the MICs of the isolates and interpret the results based on predetermined cutoff points. For example, in a study investigating *L. monocytogenes* from food and the food environment in Brazil, all 82 isolates were classified as having reduced susceptibility to BC, as their MICs varied from 16 to 128 µg/mL and the predetermined cutoff point was 10 µg/mL [34]. In another study, out of 77 *L. monocytogenes* from meat-processing facility, 17 were considered to be resistant to BC. The cutoff point of 12.5 µg/mL was applied based on the differences between MICs achieved in that study [35].

However, protocols other than assessing MICs are also applied. For example, an analysis of the sensitivity of isolates from six different turkey-processing plants in the United States was performed by spotting a bacterial suspension onto a blood agar containing BC at a concentration of 10 µg/mL. Based on this assay, 57 out of 123 isolates were identified as resistant [36]. A similar methodology was used to determine the sensitivity of 287 *L. monocytogenes* strains isolated from fish, fish products, and food-producing factories in Poland. Namely, bacterial suspensions were spotted on blood agar containing 5, 10, and 20 µg/mL of BC. Strains were classified as resistant if confluent growth was recorded on agar containing ≥10 µg/mL of BC. Based on the analysis, 40% of the isolates were considered resistant in that study [33].

In general, the common occurrence of isolates with reduced sensitivity to BC among *L. monocytogenes* originating from food products and processing environments has been observed by many authors. Although our results are not related to any particular concentrations, differences in the sensitivity of the collected isolates to BC were found. A high initial concentration of BC (6 mg/mL) allows the disinfectant to diffuse and create a concentration gradient in the agar medium. The achieved clearing zones enabled straightforward differentiation of the collected isolates.

In terms of the virulence of food-associated *L. monocytogenes* isolates, similarly to the prevalence of QACs resistance-related genes, it varies depending on the analyzed collection. The collection of the isolates analyzed herein was already studied in a publication in which their virulence potential was assessed based on the presence of genes of interest. The paper showed that all 153 isolates harbored a variant of 12 virulence-associated genes, including all from LIPI-1; four internalins, including *inlA*-*inlB* locus; and others [28]. Hence, the presence of *ilsA* from LIPI-3 was the only analysis included herein, and it allowed the virulence potential of the isolates to be differentiated to some extent. The prevalence of LIPI-3 in *L. monocytogenes* genomes varies greatly depending on the analyzed sample, sometimes even reaching 100% of isolates [37,38]. However, in the vast majority of papers, the results fall between 5% and 50% [6].

In conclusion, the prevalence of particular QACs resistance-related genes, as well as the prevalence of LIPI-3 in genomes of *L. monocytogenes,* are different in every publication, as every collection originates from different sources, different geographical areas, and is gathered during different time frames. All published results add up to a more complete picture, which allows for the tracking of global trends in terms of the prevalence of particular genes. Due to the frequent use of QACs in the food industry, genes responsible for QACs resistance are common among *L. monocytogenes* originating from food products and production environments. Based on the results published herein, such conclusions can also be drawn.

## 4. Materials and Methods

### 4.1. Isolates

A collection of 153 *L. monocytogenes* isolates was used in this study. Isolates originated from Polish meat processing plants (45 environment isolates) and meat products (108 isolates). Food isolates were isolated from bacon (51 isolates), chicken meat (21 isolates), sausage (11 isolates), smoked fish (10 isolates), and other sources (15 isolates), including, e.g., beef, pork, and fish. The isolates were collected over a period of 13 months, from October 2020 to November 2021. Isolates preserved as glycerol stocks stored at −80 °C were used for phenotypic analyses, and their DNA was used for genetic analyses. Detailed information about the collection process and DNA extraction procedure has already been published [39].

### 4.2. Phenotypic Analysis

The sensitivity of the collected isolates to BC was assessed using an agar diffusion method similar to a well-known disc diffusion method commonly applied to antibiotic sensitivity testing. However, the substance was placed directly onto the agar medium in the form of a drop of the tested solution without using discs containing the tested antimicrobial agent. This technique has already been described in the literature in the context of testing the sensitivity of *Listeria* spp. to bacteriocins [40]. The detailed methodology applied for this study is described below.

Isolates preserved in the form of glycerol stocks stored at −80 °C were used to inoculate brain heart infusion (BHI; Oxoid, Warsaw, Poland) agar medium with a sterile microbiological loop. Inoculated plates were then incubated at 37 °C for 16 h and stored at 7 °C for up to three weeks (21 days) for further use. A single colony was picked from the agar plate and used to inoculate 5 mL of BHI broth, which was then incubated at 37 °C for 18 h. After incubation, the culture was centrifuged (5 min with approx. 1700 g-force) and re-suspended in sterile deionized water (2.5 mL). This suspension was used to establish a 0.5 McFarland density in 3 mL of sterile deionized water, in which a cotton swab was then immersed and used to surface-inoculate a sterile BHI agar plate. The inoculation was performed by gently rubbing the whole agar surface in three directions.

A 10 µL drop of water-dissolved and filter-sterilized BC (Sigma-Aldrich, Poznań, Poland) solution with a concentration of 6 mg/mL was placed on the inoculated agar surface using an automatic pipette. A drop of water (10 µL) was applied as a negative control on the same petri dish, at a distance from the BC drop. Plates were left at room temperature (22 °C) for an hour to allow for the absorption of the drops into the agar medium. Then, plates were incubated at 37 °C for 18 h. After incubation, the diameter of the clearing zones around the spot of BC solution drop placement was measured in three technical replicates, and the average diameter was calculated. The experiment was performed in two independent replicates. The final result of this assay is an average diameter from two replications, expressed in mm and rounded out to the nearest integer. Sterility controls were performed with every batch of samples.

### 4.3. Genetic Analyses

Genetic analyses were performed with PCR using primers described in the literature [12,32,41,42]. Three genes associated with resistance and reduced sensitivity to QACs were analyzed, as well as the *ilsA* gene, the first gene on the LIPI-3. Primers used in the study were synthesized to order by Genomed S.A. (Warsaw, Poland). PCR reactions were performed in a T-Gradient thermocycler (Biometra, Göttingen, Germany) with the conditions given below in Table 3. The genes *ilsA*, *emrC*, and *bcrABC* were analyzed in reactions using 0.2 U RUN polymerase (A&A Biotechnology, Gdańsk, Poland), with dedicated buffer, 0.2 mM nucleotide mix (A&A Biotechnology), and 0.5 µM of primers. The gene *qacH* was detected using StartWarm HS-PCR Mix (A&A Biotechnology) with 1.0 µM of primers. Matrix DNA was added in the amount of 10 ng, whereas in negative controls, water was used instead of the matrix DNA. Reactions were performed in 10 µL of final volume.

PCR products were separated in agarose gels and visualized with ethidium bromide. One randomly chosen sample for each gene was purified using the Clean-Up Concentrator kit (A&A Biotechnology) and sequenced by Genomed S.A. company. The sequences were then analyzed using BLAST 2.15.0 [43,44].

### 4.4. Data Analyses

Data were analyzed using MS Office Excel 2019 Software. The chi-square test was used to verify the significance of data independence.

## Figures and Tables

**Table 1:** Classification of the response of the isolates to BC based on the agar diffusion analysis.

Response to BC	Number of Isolates (%)
Sensitivity	71 (46.4%)
Intermediate	3 (2.0%)
Reduced sensitivity	79 (51.6%)

**Table 2:** Summary of gene profiles prevalence in analyzed collection.

Gene Profile				Number of Isolates (%)
*emrC*	*bcrABC*	*qacH*	*ilsA*	
−	−	−	−	69 (45.1%)
+	−	−	−	59 (38.6%)
−	−	−	+	15 (9.8%)
−	+	−	−	5 (3.3%)
+	−	−	+	3 (2.0)%
+	−	+	−	1 (0.7)%
+	+	−	−	1 (0.7)%

**Table 3:** Detailed information about PCR conditions.

Gene	Primers	Amplicon Size [bp]	Cycling Conditions	Reference
*bcrABC*	F: CATTAGAAGCAGTCGCAAAGCA R: GTTTTCGTGTCAGCAGATCTTTGA	1100	94 °C 5 min; (94 °C 30 s; 57 °C 50 s; 72 °C 60 s) × 30; 72 °C 5 min	[12]
*emrC*	F: TTATTCCATTTTATTACTGGCAATG R: CGTATTTATATTTAACACTAGCCA	387	94 °C 2 min; (94 °C 15 s; 50 °C 30 s; 72 °C 30 s) × 36; 72 °C 5 min	[41]
*qacH*	F: ATGTCATATCTATATTTAGC R: TCACTCTTCATTAATTGTAATAG	366	95 °C 5 min; (95 °C 25 s; 48 °C 40 s; 72 °C 40 s) × 35; 72 °C 5 min	[32]
*ilsA*	F: CGATTTCACAATGTGATAGGATG R: GCACATGCACCTCATAAC	280	94 °C 5 min; (94 °C 30 s; 52 °C 30 s; 72 °C 60 s) × 30; 72 °C 5 min	[42]

## Data Availability

The original contributions presented in the study are included in the article (and Supplementary Material), further inquiries can be directed to the corresponding authors.

## Supplementary Materials

The following supporting information can be downloaded at: https://www.mdpi.com/article/10.3390/antibiotics13080749/s1.
